# A new role for spinal manual therapy and for chiropractic? Part I: weaknesses and threats

**DOI:** 10.1186/s12998-024-00531-6

**Published:** 2024-03-26

**Authors:** Søren Francis Dyhrberg O’Neill, Casper Nim, Dave Newell, Charlotte Leboeuf-Yde

**Affiliations:** 1 Medical Spinal Research Unit, Lillebaelt Hospital, Spine Centre of Southern Denmark, University Hospital of Southern Denmark, Østre Hougvej 55, Middelfart, Denmark; 2 Department of Regional Health Research, University of Southern Denmark, Odense, Denmark; 3 Center for Muscle and Joint Health, Department of Sports Science and Clinical Biomechanics, University of Southern Denmark, Odense, Denmark; 4 AECC University College, Bournemouth, UK

**Keywords:** Chiropractic, Chiropractic history, Professional identity, Professional development, Spinal manipulation

## Abstract

Spinal manual therapy is central to chiropractic history, clinical practice, and professional identity. That chiropractors have developed an expertise in this domain has provided some considerable advantages. However, we contend it is also at the crux of the ideological schism that fractures the chiropractic profession. In this article, which is the first in a series of two, we discuss chiropractors’ understanding and use of spinal manual therapy and do so with particular emphasis on what we see as weaknesses it creates and threats it gives rise to. These are of particular importance, as we believe they have limited the chiropractic profession’s development. As we shall argue, we believe that these threats have become existential in nature, and we are convinced that they call for a resolute and unified response by the profession. Subsequently, in part II, we discuss various strengths that the chiropractic profession possesses and the opportunities that await, provided that the profession is ready to rise to the challenge.

## Background

### Spinal manual therapy and the chiropractic profession

From the outset in 1895, the chiropractic profession has been inextricably linked to the practice of spinal manual therapy (SMT), or *spinal adjusting* as it is also known in chiropractic nomenclature. A testament to this link is the semantic similarity of *chiropractic* from Greek and *manipulation* from Latin, which have similar meanings as that which is done skillfully by hand. In this and the following paper, we use *spinal manual therapy* and *spinal adjusting* synonymously to refer to any procedure that applies mechanical forces to the spine to obtain clinical effects, i.e., not only high-velocity, low-amplitude manipulation.

Spinal manual therapy is the treatment most commonly provided by chiropractors [1–6]. Recently, we found that patients receive from one to as many as twelve SMT procedures in a single consultation [7]. We contend that both those within and outside the profession very closely associate chiropractic with the practice of SMT, and for good reason. It is not unreasonable to ask whether chiropractic and SMT are, in fact, synonymous in the minds of the public. It is also reasonable to ask, whether chiropractors themselves have impressed the centrality of SMT on patients, with the result that the public assumes “that’s what chiropractors do”. Spinal manipulative therapy obviously plays a central role in the profession’s image and distinct identity. No doubt, this has historically helped the chiropractic profession stand out, but it has also caused it to stand apart.

The issues concerning chiropractic identity and philosophical divides have been examined in several publications, for instance, by Meeker and Haldeman in 2002 [8], Reggars in 2011 [9], Schneider et al. in 2016 [10], Brosnan in 2017 [11], Leboeuf-Yde et al. in 2019 [12], and many others.

In the following text, we shall approach such issues from the angle of the unique position that SMT holds in the profession. We will describe how a narrow focus on a single modality combined with controversial theories and unrealistic expectations of its clinical efficacy have contributed to the precarious position we believe the profession now finds itself in. In this paper, we shall specifically concentrate on what we consider are the major weaknesses of this situation and the threats it poses for the future development of the chiropractic profession. In isolation, the present paper may thus seem overly critical or abrasive to practitioners of SMT, as it focuses on negative issues– i.e. threats and weaknesses. Keep in mind, that in the following paper will shall discuss positive aspects under the heading of strengths and opportunities in relation to SMT. These two papers are complimentary, as any frank discussion of the issue necessitates dealing with the negative and the positive.

### Overview of the chiropractic landscape

A number of chiropractors promulgate simple explanations steeped in historical dogma for patients’ problems, such as: *vertebral subluxations exist, they are bad for you in any number of ways, chiropractors can find them and fix them and that is good for you*. By contrast many other chiropractors subscribe to a limited paradigm of chiropractic as musculoskeletal (MSK) evidence-based medicine/healthcare (EBM), in which SMT nonetheless, plays a lead role [13–15].

In light of this diversity of clinical paradigms, the chiropractic profession fails to project a coherent image to the greater public beyond the use of SMT in the management of spinal pain disorders, and it has very different legal and professional standing in different jurisdictions, which confounds the issue.

We expand upon these observations in the following.

### Weaknesses

#### Solid foundations or a burning platform?

As an old and well-established healthcare profession, chiropractors obviously hope that the profession is seen as standing proud and strong. However, we fear many in the profession are oblivious to the burning platform on which, we are convinced, it stands. We are not so much referring to attrition of new colleagues, diminishing business prospects or an oversupply of graduates [16] because such things are likely to vary considerably between countries and over time. Of greater concern to us is a disconcerting professional image [17–20], persistent and common claims of providing care for non-MSK disorders [21–23], internal conflicts which run deep and remain unresolved [12, 24], professional development and integration into mainstream healthcare, which in our perspective has not exactly progressed at speed. At the core of this is a potentially more damaging issue: Relevant science is raising serious questions about original chiropractic dogmas [25], and conversely, dogmatic chiropractors are seriously questioning science as relevant [9, 26]. It really does seem to us that there is a critical schism within the profession, which goes beyond the continuous navigation between scientific evidence and clinical individualization, which is common to all healthcare.

All of this, we argue, constitutes a burning platform for the profession, which is intricately tied to the position that SMT holds in the chiropractic professional identity and paradigm.

### The burden of the subluxation dogma

Interestingly, many historical quotes by chiropractic pioneers attempting to define the profession did not focus on SMT or MSK disorders. For instance, the founder of the chiropractic profession, Daniel David Palmer, is quoted as saying: “Life is the expression of tone. In that sentence is the basic principle of Chiropractic.” and “Chiropractic is a science of healing without drugs.” His son, Bartlett Joshua Palmer, went on to say: “The power that made the body, heals the body.”, “Nature needs no help, just no interference.” and “So what Chiropractic does, is that it simply ‘takes the handcuffs off Nature’, as it were.”

Despite being nearly a century-and-a-half old, such traditional chiropractic concepts are still quoted or paraphrased by some chiropractors [27–31] and by some chiropractic organizations [32] to describe what they perceive as contemporary and central chiropractic tenets. Thus, the literature and the internet abound in such quotes from early and contemporary chiropractors, which revolve around similarly lofty metaphysical concepts.

However, such statements define no scope of practice, clinical armamentarium, ethical standards, organizational emplacement, or academic or educational requirements– i.e., none of the elements which normally define a profession [33]. Nor do they explicitly focus on SMT. So how did SMT come to take centre stage in chiropractic professional identity?

The centrality of SMT in chiropractic identity stems from a line of reasoning derived from the elusively defined vertebral subluxation complex and its relationship to the lofty metaphysical concepts mentioned above. Briefly, the subluxation complex is theorized as a mechanical joint lesion, which impedes health and is remediable by spinal adjusting, i.e. SMT.

A quick internet search will reveal that some chiropractors suggest the ramifications of such subluxations range from local pain to organ disease and even early death [34]. With the subluxation as a theoretical basis, SMT can thus be elevated from ‘*just*’ a mechanical procedure to a purported panacea that will remove the root cause of all manner of minor and serious health issues and even bring about a life of “full potential” to those who are healthy.

The failure of parts of the profession to recognize that clinical practice rooted in historical dogmas, by its very nature, is detrimental to professional development is central here. This is because it runs counter to the principles of evidence-based healthcare and all but ensures that outmoded concepts and inappropriate clinical practices persist beyond their sell-by-date. Using different terminology, such as ‘spinal adjustments’ and claiming them superior to SMT, will not magically make it otherwise.

### Unlimited clinical indications for SMT

Rooted in the historical subluxation paradigm, many chiropractors have had an optimistic view of just how broad a spectrum of health problems they were able to prevent and cure with SMT, and it seems clear to us that this is still the case for at least some chiropractors [1, 35, 36]. However, the best available scientific evidence does not support such an optimistic view [37–39].

Thirty-seven per cent of Australian chiropractors report providing subluxation based care ‘Always’ or ‘Most of the time’ [40]. In North America, 21% reported being subluxation-based, and a further 22% regarded themselves as providers of “holistic primary care” [15]. Furthermore, three out of four American chiropractors [41] and eight out of ten French chiropractors [42] have been reported as favouring or advertising a broad scope of practice including non-MSK disorders. Similar findings are reported in the United Kingdom [36]. Finally, in a survey of European chiropractors, 25% self-categorized themselves as treating biomechanical/organic-visceral complaints or subluxations as an obstruction to human health. However, the proportions were substantially different across different countries [14]. In Belgium, only 38% regarded their scope of practice as being restricted to neuro-musculoskeletal disorders, and 58% would treat visceral complaints [1].

Yet, the vast majority of patients seek out chiropractic services precisely for MSK disorders [3, 5, 43]. It is notable therefore, that there exists a discrepancy between some chiropractors’ expectations and the public’s perception of the relevance of using SMT for non-MSK disorders [19, 44, 45].

The scientific evidence for SMT treatment of non-MSK disorders is absent [39], and as outlined above, the public’s perception of the relevance of chiropractic is predominantly restricted to MSK disorders. Furthermore, promulgating such a broad scope of practice will likely foster criticism, a loss of professional esteem, and decreased cultural legitimacy [17, 18, 22]. The irony here is that MSK conditions are hugely common, disabling, costly to society [46, 47], and often inappropriately managed [48]. Thus, chiropractors could easily keep themselves busy providing safe and relevant care for MSK disorders without extending their services to other more controversial clinical areas. We shall discuss this further in the second paper of this series.

It is difficult to know precisely how the different surveys on non-MSK disorders, subluxation-based paradigms and holistic primary care map onto each other, but seen in light of the historical chiropractic paradigms, it suggests to us, that a subgroup of chiropractors continue to see chiropractic as relying primarily on SMT and as relevant for non-MSK disorders. By contrast, the public and other health professionals generally do not.

In summary, we purport, that providing SMT for non-MSK disorders probably does nothing beneficial for patients, but harms the profession’s image and standing in society and negatively affects its legitimacy in the wider healthcare landscape.

### Over-reliance on SMT

Many chiropractors do not believe that SMT constitutes a panacea or is relevant for non-MSK disorders. However, even when they provide SMT for MSK disorders, such as low back pain (LBP) in line with 81% of clinical guidelines [49], for many it seems that SMT assumes a more prominent role than it reasonably should. There are two issues here: Firstly, the science of the last few decades suggests clearly that MSK conditions like LBP are generally not cured in the long term but commonly have intermittent or chronic fluctuating trajectories [50]. Secondly, the long-term perspective suggests that such conditions need to be appropriately *managed*, i.e., it is rarely a case of finding the right *treatment* to cure the issue once and for all [51]. Spinal manual therapy is often relevant, but over-reliance on just one treatment modality will distract from other relevant clinical considerations, which necessarily need to be considered in the long-term management strategy of such multi-factorial health problems.

Importantly, in cases where chiropractors practice in accordance with an MSK-only paradigm, many have an unrealistic expectation of their ability to change the natural course of the condition. For instance, a large multi-centre study in Norway showed that the chiropractors assumed that 80% of their patients had experienced no new problems in the 12 months after chiropractic care. In fact, 74% of their patients reported having had such problems [52]. In another cohort of chiropractic patients with low back pain, only 20% could be classified as making a sustained recovery from pain over a six-month period [53], which would probably surprise many chiropractors.

Having too high expectations of the effects of treatment (SMT and otherwise) is not a misconception exclusive to chiropractors. However, after having dealt with the initial acute complain, there is no good reason to promise patients that SMT can get to the bottom of things or find the *root cause*, an often-heard claim among some in the profession.

We, therefore, conclude that chiropractors rely too heavily on SMT in the management of MSK disorders at the expense of other relevant clinical activities. It ought to constitute only one element in a larger orchestra of clinical instruments.

### Over-engineering of SMT

Several names have been given to the ‘manipulable lesion’, which is the target of SMT, such as ‘vertebral subluxation complex’, ‘facilitated segment’, ‘biomechanical dysfunction’, ‘spinal boo boo’, and many others [54]. The lesions are purported to be detectable, e.g. by static and dynamic palpation, x-ray analyses, dynamic testing of skeletal muscles, differences in superficial skin temperature, and many other means [55].

The appropriate way to apply SMT for the correction of such lesions also varies greatly. Some techniques rely on sustained pressure by gravity alone, some employ a thrust, some apply sustained post-thrust pressure, some a recoil, some are applied as an assisted and some as a resisted force, some use mechanical instruments, others are done by hand, and so on [54, 56].

Although this, on the face of it, provides a richness of approach, we contend that it has potentially trapped the profession in an eternal and fruitless search for the *correct* way to identify the lesion and the *best* technique to fix it. While other healthcare professions have moved towards a multi-faceted approach, many in the chiropractic profession, it seems to us, place little emphasis on other treatments and approaches to care, including non-physical elements within the therapeutic encounter, remaining instead rather preoccupied with the technical particulars of how to find it and fix it.

There is some research into such topics but no good scientific evidence that supports the supposition that technical details are important for clinical outcomes. Indeed, deciding which vertebral level to manipulate, let alone how, appears to be of limited relevance [57].

Leaving the science aside, it is also worth contemplating, why such technical specificity in SMT has not resulted in a clinical streamlining of SMT techniques. If, indeed, it does make a noticeable difference for clinical outcomes, one might have expected clinicians to notice and converge on the more effective procedures. This is not the case.

In any case, the enormous societal challenges and individual consequences posed by MSK pain will not be solved by a change in thrust vector or degree of amplitude, for which reason the extreme emphasis on the ‘technique’ may well be excessive. Arguably, the time and effort afforded to excessive minutiae of SMT technicalities, in both education and clinical consultation, poses a problem: It shifts focus and resources that could have been invested into other aspects of the complex and multi-factorial problem that MSK disorders present.

### SMT as a threat to chiropractic

The baggage of historical dogma still weighs heavily within the chiropractic professional view of SMT. Thus, it is offered in clinical contexts for which there is no evidence for its relevance, it is often overused in contexts where it is relevant, and it consumes an unnecessary amount of attention to technical detail– all at the expense of other clinical considerations. This is apparent not only in much of clinical practice but also in the education of chiropractors, where a plethora of adjustive techniques are taught, whilst other evidence-based approaches are sometimes lacking (e.g., exercise) [58].

### Threats

One would expect that threats to a profession would come from the outside. However, the weaknesses we identified have their origins *within* the profession. In other words, we suggest that threats to the chiropractic profession are largely self-inflicted.

### Science and the impact of evidence based medicine

As outlined in the preceding sections, the evidence leaves much to be desired as far as demonstrating that vertebral subluxations exist, that they are bad for you, that chiropractors can find them and fix them, or that the treatment has the extensive health benefits postulated by some. This entire chain of traditional conjectures, thus, makes for a very precarious foundation on which to build a profession, especially so in an age of Evidence Based Medicine (EBM).

An EBM approach to health-care does not imply that scientific evidence should rule absolute at the expense of all other considerations: Patient preferences and clinical expertise also play important roles in balanced clinical decision making [59, 60]. Notwithstanding, it is not a carte blanche to ignore a growing body of science relevant to clinical practice and theoretical constructs. Any notion that historical chiropractic dogmas or a different vocabulary somehow shield the profession from the bright light of science is illusory and naïve.

Although the profession claims to embrace a scientific approach to the practice of chiropractic, in general, including the use of SMT, we suggest it does not always seem to do so convincingly. If such a commitment is not seen as wholehearted and universal, it will be difficult to sway those who already hold a sceptical view of our profession [17–19]. This applies at all levels, from private practice to policymakers. When the chiropractic umbrella organization, the World Federation of Chiropractic (WFC), advice chiropractors to “*refrain from any communication that suggests spinal adjustment/manipulation may protect patients from contracting COVID-19 or will enhance their recovery*” [61], they are seen to promote EBM and responsible clinical practice. However, when the WFC also “*champions the rights of chiropractors to practice according to their training and expertise*”, it is effectively attempting to have it both ways [62].

Committing to scientific accountability is a double-edged sword and means accepting the best available evidence on SMT, whichever way it points and aligning clinical practice to such evidence, even if it goes against traditional formative theories. Presently, we fear that a substantial faction of the chiropractic profession is not ready to move wholeheartedly in this direction.

Science and EBM thus threaten the traditional chiropractic relationship with SMT in two ways: Firstly, there is no avoiding a genuine commitment to science and EBM if the profession wants to remain relevant– failure to do so will result in ostracization from academia and the established healthcare system. Secondly, once committed, the profession must follow the best evidence regarding SMT wherever it leads us. To some in the profession, this poses a threat to the kind of chiropractic practice that treats historical subluxation theories as legitimate tenets. It may also present a threat to those who rely primarily on SMT in the management of MSK disorders at the exclusion of other treatments or approaches, in so far as EBM suggests they are relevant.

### Accountability to authorities and 3rd-party payers

Healthcare coverage varies greatly between countries and settings. It is often a combination of out-of-pocket-expenses, national health coverage, and private or employer-financed health insurance. Whether public or private, third-party payers will be motivated to keep expenditures low and will look to provide services that are well-described, standardized and aligned with evidenced clinical guidelines.

Funding by third-party payers can, thus, be seen as a threat to the traditionalist segment of the profession, as it opens for a degree of scrutiny of clinical practice tied to financial reimbursements. In other words, third-party coverage of SMT may be restricted to more specific indications than vertebral subluxation.

### Internal conflicts are glazed over and left to fester

The chiropractic profession has been conflicted about its identity and role from early on. A recent paper likened the profession to a dysfunctional marriage and asked whether the time was ripe to pause and consider the prospects of disparate groups staying together versus parting ways [12].

This internal conflict has been tolerated for a century, but has always been a source of internal tension and external criticism. The widespread adoption of EBM principles reflects a general move away from authority-based to science-based health-care, which means that the internal schism in chiropractic is becoming increasingly challenging to glaze over. Although the internal conflict in chiropractic has always been a problem, we believe it has now become an existential threat, especially for those that subscribe to EBM and an MSK-limited scope of practice. How this internal conflict relates to the indications for and use of SMT has been described above.

### Isolated by choice

Not only is the healthcare system/market changing from what some have termed *Eminence Based Medicine* towards *Evidence Based Medicine*, i.e., from professional authority to a scientific basis, it is also increasingly becoming a team effort, where multiple healthcare professionals play different parts in a greater symphony– or, indeed, cacophony.

There is a strong tradition for collaboration between different medical specialties, nursing specialties, occupational and physiotherapists. Obviously, the boundaries and overlaps between these different professions are continuously being renegotiated, which can be a source of tension. Rarely however, will a medical doctor be heard arguing that there is no need for nursing at all, or a physiotherapist claiming that surgeons have no relevance in modern health-care.

For chiropractors, the situation is much less clear. Obviously, many sympathetic voices acknowledge the relevance and value of what chiropractors do or *could do*, but they are often hedged with caveats concerning the scope of practice and regarding non-MSK disorders, in particular. In general, however, other health-care professions and authorities are not going out of their way to invite chiropractors to join the multi-disciplinary party. We speculate that an important reason for that situation, is the chiropractic profession’s failure to project a unified and coherent image as mainstream MSK specialists. Furthermore, we fear that it would be very difficult for chiropractic to move forward towards greater integration, and, in some countries, it probably already missed the boat.

We acknowledge, of course, that there are exceptions, but chiropractors are a rarity in orthopedic departments, rehabilitation units, accident and emergency departments, and a host of other clinical settings where MSK disorders are managed. The same holds true for administrative and political organizations, governmental and non-governmental alike. By and large, chiropractors are to be found in private clinical practice. It is tempting to deny this by pointing to those exceptions, where chiropractors actually have made inroads into other clinical and political areas, and we do not mean to undervalue or denigrate their importance. Instead, we invite the reader to contemplate why, after more than a century, the profession can still only celebrate such cases as exceptions rather than as commonalities.

We suggest that chiropractic’s isolation is self-imposed mainly due to historical dogmas described in the preceding text, and its preoccupation with SMT, and the impact these have had on chiropractic’s image. We would argue that such isolation obviously threatens the profession’s continued existence.

### Competition

Other health-care providers, including physicians, osteopaths, and physiotherapists, also competently provide SMT. Thus, the mainstay of chiropractic clinical services is under considerable competitive pressure from other professions. We suspect this has expedited the development of specific terminology, like ‘chiropractic adjustment’, in the hope that this would build a security wall around the profession to keep competition out. In some jurisdictions, this has taken the form of trying to legally restrict other professions from providing SMT [63].

In reality, though, there is no reason to expect meaningful technical or clinical differences between providing SMT versus ‘chiropractic adjustments’. The only difference can be found in the underlying intention and theoretical framework, within which the treatment is given. Those are, of course, factors that reside inside the head of the chiropractor and not in the spine of the patient. Notwithstanding contextual factors [64], expecting different outcomes from the same intervention in the same conditions solely based on intent or theoretical framework smacks distinctly of magical thinking [65], which is conducive neither to inter-professional collaboration nor evidence-based practice.

Abandoning traditional subluxation theories could arguably constitute a threat to some chiropractors, as the difference between different providers of SMT becomes washed out unless of course there are other differences of importance. Conversely, insisting that SMT provided by chiropractors is fundamentally different because of the language and theories that go with it, is also a potential threat.

## Conclusion

As we have argued, the chiropractic profession is weighed down by the burden of historical theories regarding SMT, which, for some in the profession, have all the characteristics of dogmatic articles of faith. In our opinion, the unlimited scope of practice, which is still advocated by some chiropractors, and which has not been met with unequivocal political rejection, an over-reliance on SMT in the management of MSK disorders, and an over-emphasis on the technical intricacies of SMT represent weaknesses within chiropractic. We argue that these are obstacles to professional development and the major causes of professional stagnation both intellectually and in the market place.

We also discussed what we consider to be *threats* to the chiropractic profession. Science, the impact of EBM, and accountability to authorities and third party-payers all pose threats to the traditional chiropractic paradigm and, thus, to those within the profession, who practice within such a paradigm. In the marketplace, competition from other professions that provide care of patients with MSK disorders, including SMT, and are better positioned to be integrated into the wider health-care system/market represent a threat. Moreover, finally, the internal schism in chiropractic represents a threat to professional development, as it prevents the profession moving forward in unison with a coherent external message.

We have described those weaknesses and threats, knowing full well, that we do so from our perspective of chiropractic as EBM with a limited MSK scope of practice, i.e. from outside the subluxation frame of reference.

We recognize that for those who look at SMT from the perspective of traditional, subluxation-based chiropractic, things will look very different: What we identify as weaknesses may be seen by others as the pillars of chiropractic practice, and what we see as threats could appear as just peripheral and ephemeral distractions to the enduring core of chiropractic ideas. Such is the character of the schism at the heart of chiropractic.

None-the-less, having described what we identify as serious weaknesses and threats arising from the profession’s relationship to SMT, it has not escaped our attention that it also gives rise to several strengths, which serve the profession and its patients well. In turn, it follows that a number of opportunities are presenting themselves for the future of SMT and chiropractic. We shall discuss these in Part II of this paper.

## Data Availability

Not applicable.

## Abbreviations

SMT: Spinal manipulative therapy
MSK: Musculoskeletal
EBM: Evidence-based Medicine
